# Development and differentiability of three brief interventions for risky alcohol use that include varying doses of motivational interviewing

**DOI:** 10.1186/s13722-017-0102-0

**Published:** 2018-02-27

**Authors:** Jennifer E. Hettema, Stephanie A. Cockrell, Abigail Reeves, Karen S. Ingersoll, Paula J. Lum, Richard Saitz, Cristina M. Murray-Krezan, Valerie A. Carrejo

**Affiliations:** 1 Department of Family and Community Medicine, University of New Mexico, Albuquerque, NM USA; 2 Department of Psychiatry and Neurobehavioral Sciences, University of Virginia, Charlottesville, VA USA; 3 Department of Medicine, University of California San Francisco, San Francisco, CA USA; 4 Department of Community Health Sciences, Boston University School of Public Health, Boston, MA USA; 5 Department of Internal Medicine, University of New Mexico Health Sciences Center, Albuquerque, NM USA

**Keywords:** Motivational interviewing, Brief alcohol intervention in primary care, Treatment fidelity, Differentiable interventions, Risky alcohol use

## Abstract

**Background:**

While brief intervention (BI) for risky alcohol use generally yields positive effects among those identified by screening, effect sizes are small and there is unexplained heterogeneity in outcome. The heterogeneity may be related to differences in intervention style and content, including elements of motivational interviewing (MI). To date, it has been difficult to interpret the role of MI in BI and these gaps in knowledge interfere with efforts to train, disseminate and implement BI that retains and maximizes efficacy. This study sought to develop BI protocols with varying doses of MI and test their differentiability. Differentiable BI protocols could allow for future studies that prospectively evaluate the role MI plays in affecting BI outcome.

**Methods:**

We developed three intervention protocols: brief advice, standard BI (NIAAA Clinician’s Guide), and MI-enhanced BI and administered them to 45 primary care patients who reported exceeding recommended drinking limits. We then rated the BI sessions for fidelity to the assigned protocol as well as MI consistency based on Motivational Interviewing Treatment Integrity (MITI) scale scores. The differentiability of BI protocols was determined by calculating fidelity to assigned protocols and comparing MITI scores using pairwise, Tukey-adjusted comparisons of least squares mean scores.

**Results:**

High rates of fidelity to each protocol were achieved. The three BI protocols were also highly differentiable based on MITI scores.

**Conclusions:**

The three interventions can be used in future trials to prospectively examine the role MI has in determining BI outcome.

*Trial registration* clinicaltrials.gov NCT02978027, retrospectively registered 11/28/16

**Electronic supplementary material:**

The online version of this article (10.1186/s13722-017-0102-0) contains supplementary material, which is available to authorized users.

## Background

Screening and brief intervention (SBI) for risky alcohol use in primary care settings has demonstrated efficacy. In a meta-analysis of 23 controlled studies, patients identified by screening in primary care settings who received BI consumed less alcohol on average (3.6 fewer drinks per week) and were less likely to exceed limits (12% fewer) than control patients at one-year follow-up [1]. However, despite overall positive findings, there are high levels of variability in BI effects, with significant unexplained heterogeneity in outcomes [1–3]. In fact, the number of studies that do and do not find significant BI effects are nearly evenly split [4]. This variability may be attributable in part to differences in the intervention components and communication style that underlie tested BI protocols, including fidelity to motivational interviewing (MI).

MI is a patient-centered communication style that seeks to draw out and enhance patients’ own motivation to change health behaviors [5]. Studies of SBI frequently report that the tested BI protocol was conducted in a manner that was consistent with an MI approach, but additional methodological details are often lacking. For example, Richmond et al. [6] briefly mention that “Patient counseling included techniques of motivational interviewing,” Senft et al. [7]. mention that research staff “were trained in principles of motivational interviewing,” Fleming et al. [8] included a workbook “based on the principles of motivational interviewing,” and Reinhardt et al. [9] provided counseling sessions “based on motivational Interviewing,” in which “the main content of each intervention was the enhancement of motivation.” None of these examples provide a specific description of how MI was incorporated into the intervention.

There are several potential ways in which MI principles or skills could be integrated into BI. For example, interventionists could be trained to take a collaborative or empathic stance, to avoid potential roadblocks to communication such as labeling and unsolicited advice, to use specific frequencies of communication skills such as complex reflections, to conduct specific exercises, to adhere to different decision rules regarding patient readiness levels, or some combination of the above. Lack of methodological details regarding the content and structure of brief interventions makes it difficult to assess the moderating impact of MI dose on outcome across trials, and primary care-based meta-analyses to date have been unable to report on the impact of this construct [1–4].

Although MI has been found to be effective for addressing alcohol issues in medical care settings [10] and aligns with several principles of medical ethics [11], there are many barriers to implementing MI in brief medical interactions. Healthcare providers are faced with many competing priorities and limited time with patients, creating pressure to conduct preventive and behavioral health interventions as efficiently as possible [12]. Conducting MI, even its briefest applications, is more time consuming than simple advice giving. In addition, MI is a complex set of skills that requires flexible application and contingent responding to moment-to-moment changes in what patients say [5]. Proficiency in the approach requires extensive training time, ongoing supervision and resources that are often limited among medical trainees and clinicians. A recent review of MI training for healthcare professionals found that average training duration exceeded 1 day and in many cases such training failed to yield clinical improvements in MI proficiency [13]. Lastly, medical clinicians themselves often perceive barriers to implementing MI, including concerns about lack of time, low levels of perceived responsibility, low confidence, and lack of support [14].

To date, there is a dearth of empirical work examining mechanisms of behavior change in SBI for risky alcohol use [15]. Of the available research that has focused on the impact of MI within SBI, results have yielded mixed findings [16]. For example, Bertholet et al. [17] re-analyzed combined data from three controlled BI trials for proficiency in important MI behaviors and found no consistent predictors of outcome. In a similar secondary analysis designed to test the mediating link between MI-consistent behaviors such as open-questions, affirmations, and reflections, client change talk, and outcome proposed by the prevailing MI model [18], this hypothesized causal chain was only supported in specific conditions, such as with therapists who had more than 3 years experience [19].

There have been some attempts to compare different SBI approaches prospectively. For example, the Effectiveness of Screening and Brief Alcohol Intervention in Primary Care Trial (SIPS trial) compared three brief intervention conditions for hazardous drinkers in primary care settings: an informational leaflet, a brief advice session, and an MI-informed brief lifestyle counseling session [20]. Results of the SIPS showed no significant differences in drinking reduction between the three conditions at follow-up, although patients in the lifestyle counseling group had significantly increased readiness to change and satisfaction with the intervention. While this trial did compare different intensities of intervention, it was confounded by content and not specifically designed to allow for inferences regarding the role of MI strategies on outcome, as treatment modalities were additive, with all participants first receiving brief advice. Additionally, the content and structure of brief advice was not tightly controlled and study authors report that study interventionists, who were clinic-based providers, often extended into more therapeutic tasks.

More work has been done with regards to alcohol treatment, which may or may not be generalizeable to SBI in primary care. Here, findings are mixed, with some studies supporting the use of specific MI structure and content, and others not. For example, Morgenstern and colleagues attempted to dismantle the active components of MI treatment by prospectively comparing 4-sessions of MI, to non-directional MI, to a no treatment control condition and found similar outcomes across condition [21]. On the other hand, Field and colleagues compared brief advice, brief MI, and brief MI plus a feedback based telephone booster among trauma center patients who recently consumed alcohol and found that the BMI plus booster condition outperformed other treatments [22]. Apodaca conducted a recent secondary analysis of a brief intervention for college students with problems drinking and found that therapist use of Mi microskills such as reflections and open-questions produced both change and sustain talk [23]. This trend was also observed on a recent multivariate meta-analysis of MI process and outcome, which found that both therapist microskills and global ratings predicted change and sustain talk and that sustain talk was predictive of poor outcome, providing equivocal support for the proposed MI theory of change [24].

While we have evidence to suggest that primary care-based brief advice may be just as effective as MI with smoking cessation [25], the evidence is not clear for BI for risky alcohol use. One strategy for determining the impact of MI on BI efficacy is by prospectively comparing SBI protocols with varying doses of MI content. While mechanisms of action research have revealed variability in MI within clinical trials, to date, it has not been demonstrated that is possible to create interventions that are reliably differentiable while still maintaining consistency within a general BI structure. In the current study we sought to develop SBI protocols that were differentiable in terms of dose of MI, with the goal of prospectively comparing them in a future controlled trial.

## Methods

### Study procedures

Participants were recruited during regularly scheduled primary care visits at two university-operated community primary care clinics. While the university system electronic medical record prompts medical assistants to conduct alcohol screening annually during triage at primary care appointments, there is no standardized SBI protocol in place, compliance with screening is variable, and providers do not typically follow up regarding screening results. Patients attending the clinics were approached in the waiting room or the exam room prior to their scheduled visit to determine their interest in being screened for eligibility in the research study. Interested participants were asked to fill out a paper copy of the NIAAA screening questions (“Do you sometimes drink beer, wine, or other alcoholic beverages?, “How many times in the past year have you had 5 or more (4 for women) alcoholic drinks in one day?” [26], and the Alcohol Use Disorders Identification Test - Consumption (AUDIT-C) questionnaire [27], which includes three multiple choice questions measuring drinking frequency in the past year, number of drinks consumed on a typical drinking day, and frequency of risky drinking episodes in the past year. Inclusion criteria were endorsement of past year risky drinking, defined as any score other than zero in response to the NIAAA question about drinks in 1 day. Participants with an AUDIT-C score greater than 7, which indicates a likely alcohol use disorder were excluded, given that BI may not be sufficient for them. Eligible participants met with a researcher in a private exam room to provide informed consent. During their regularly scheduled primary care appointment, consenting patients were administered a baseline assessment and BI in their exam room prior to their provider visit or in a private adjacent office following their visit depending on the clinic flow and the availability of the clinician. Participants were randomly assigned to one of three distinct intervention conditions: BI based on the approach described in the NIAAA Clinician’s Guide (NIAAA), Brief advice (BA), or an MI-enhanced NIAAA Clinician’s Guide approach (MI). After the intervention, participants received a $25 gift card.

### Baseline assessment

Following consent, participants completed a brief baseline assessment. The assessment was designed to be minimal to avoid potential assessment reactivity effects [28, 29]. Baseline assessment included a brief demographic form and a locator form to assist with later follow-up. Within the context of the SBI protocol, interventionists verbally re-administered the NIAAA heavy drinking episode screening question, determined drinking quantity and frequency, and assessed for AUDs using two questions related to drinking in harmful situations and drinking more than intended [26]. Participants who endorsed either of these criteria were further assessed for AUD by responding to questions that correspond to each DSM 5-criterion.

### Intervention protocols

Interventions were adapted from the NIAAA protocol “Helping Patients Who Drink Too Much: A Clinician’s Guide” [26]. All interventions were video recorded for later coding. Intervention protocols can be accessed in Additional files 1, 2 and 3.

#### NIAAA Clinician’s Guide (NIAAA)

The NIAAA brief intervention was adapted directly from the NIAAA publication “Helping Patients Who Drink Too Much: A Clinician’s Guide” [26]. This protocol includes both MI-consistent and MI-inconsistent elements. The protocol involves screening (see above) and then assessing for quantity and frequency using closed-ended questions. The guide has some ambiguity regarding strategies for assessing for alcohol use disorders. Although AUDIT-C inclusion criteria were designed to rule out patients with a likely alcohol use disorder, this screening tool does not have perfect sensitivity and it was still possible for patients to have an AUD upon further assessment. In this trial, assessment of alcohol use disorders was conducted using Vinson’s [30] two questions described above, instead of the entire diagnostic checklist. Those exceeding recommended limits were told: “You’re drinking more than is medically safe.” They were shown a chart describing U.S. adult drinking patterns that included recommended drinking limits and the prevalence and consequences of exceeding them. The clinician then stated: “I strongly recommend that you cut down and I’m willing to help.” The protocol called for assessment of readiness to change (“Are you willing to make changes in your drinking?”). For patients unwilling to make a change, the clinician was instructed to restate their concern, encourage self-reflection by asking the patient about reasons to cut down on drinking and barriers to change, and reaffirm willingness to help. For patients willing to make a change, the clinician was instructed to help the patient develop a plan to cut down within maximum limits, agree on specific steps and strategies, and provide a tip sheet on strategies for cutting down. The clinicians in the trial were specifically instructed to be warm and friendly. However, they were also instructed to avoid the use of MI micro-skills such as reflection, statements that infer the meaning underlying what patients have said, and not to differentially respond to change talk, by focusing on patients’ motivations (versus barriers) to change.

#### Brief advice (BA)

The brief advice protocol was designed by removing MI-consistent elements from the NIAAA Clinician’s Guide, while leaving other elements that were either not MI-consistent or neutral. The protocol involved screening and assessment using the same strategies described above. Patients exceeding recommended limits received the same feedback, information, and advice described above. All patients were then provided with a tip sheet on strategies for cutting down (e.g. pacing and spacing, keeping track, know your ‘no’) and encouraged to follow-up with a behavioral health provider with any questions or concerns. As above, the clinician in the trial was specifically instructed to be warm and friendly, but avoid the use of reflection and evocation of change talk.

#### MI-enhanced NIAAA Clinician’s Guide (MI)

The MI intervention condition was also adapted from the NIAAA Clinician’s Guide, with additional modification to include elements of MI. At the start of the protocol, clinicians were instructed to normalize SBI and ask the patient’s permission before discussing alcohol use. The NIAAA screening questions were administered as designed, which is closed-ended. Otherwise, when possible, closed questions were reframed as open questions. Assessment of quantity, frequency, and AUD symptoms was done using open questions, followed by closed questions if additional clarification was needed. The ask-tell-ask technique was used to share feedback regarding the patient’s risky drinking status and exchange information regarding U.S adult drinking patterns. Ask-tell-ask, involves asking patients what they know about a particular topic, providing information in a tailored way, and then asking the patient what they think of the information or how they might apply it. For patients low in readiness to make a change, clinicians built readiness using one of two structured MI tools: a scaling ruler or a road map. Scaling rulers involve asking patients to self-assess their perceived importance, confidence, or readiness to change and then strategically evoking change talk by inviting them to speak about why their assessment wasn’t lower. The road map exercise involves evoking change talk by asking patients to imagine their life down two paths and to speak about the good things that might happen if they change and the bad things that might happen if they don’t change. For patients high in readiness to change, the ask-tell-ask technique was used to explore the tip sheet on strategies for cutting down and develop an action plan. The MI intervention called for interventionists to use reflective listening statements and summaries throughout the protocol and differentially focus on the patient’s reasons for change.

### Fidelity and MI dose assessment

All intervention sessions were video recorded and objectively rated using two coding instruments. An independent fidelity coder rated all sessions and members of the research team co-rated a subset to establish estimates of interrater agreement. Research team members never coded their own sessions. Because of the different content and structure and each intervention, it was not possible to blind raters to condition. Fidelity checklists were developed specifically for each condition, and included yes, no, and not applicable check boxes for each step of the protocol. The total fidelity score was the proportion of protocol activities that were conducted that should have been conducted. The fidelity checklists are accessible in Additional files 1, 2, 3, 4, 5, and 6. Video recordings of interventions were also rated using the Motivational Interviewing Treatment Integrity Code 3.1.1 (MITI), a coding instrument designed to measure competence in MI that has demonstrated reliability and sensitivity [31, 32]. The MITI includes specific behavior counts thresholds, such as percent open questions, reflection to question ratio, and percent complex reflections, as well as assessment of global characteristics of the intervention, such as empathy and evocation. The MITI also has suggested benchmarks for beginning proficiency and competency.

### Interventionists

The study interventionist was primarily the second author, a master’s level social worker with extensive training in MI, however, the first author, a clinical psychologist, also conducted some sessions. Both are members of the Motivational Interviewing Network of Trainers (MINT) who completed a MINT “training for trainers (TNT).” Training for the study intervention involved extensive practice with mock patients until 95% fidelity measures were consistently met on all SBI protocols. The first author conducted bi-weekly feedback and supervision during the trial.

### Statistical analysis

To determine the reliability of fidelity coding, intraclass correlation coefficients (ICCs) were calculated using a one-way random model for absolute agreement [33]. Cicchetti has recommended that ICCs be interpreted as follows: below 0.40  =  poor; 0.40–0.59  =  fair; 0.60–0.74  =  good; and 0.75–1.0  =  excellent [34]. To determine the differentiability of the three interventions, mean percent fidelity to each protocol was compared across the three conditions using one-way ANOVA. MITI scores were compared across the three conditions using pairwise, Tukey-adjusted comparisons of least squares mean scores. To reduce the possibility of spurious findings, a Bonferroni adjustment was made for 9 comparisons. Outcome measures met normality assumptions required for these analyses.

## Results

### Interrater agreement

Of the 45 sessions, 9 (20%) were co-rated to assess interrater agreement. Rates of interrater agreement were excellent for the fidelity checklist (ICC = 0.93, 95% CI = 0.72, 0.98). All MITI global scores (evocation, collaboration, autonomy support, direction, empathy) were also in the excellent range, with the exception of direction, which could not be calculated because there was no variability in rating. All behavior count threshold (% open questions,  % complex reflections, reflection to question ratio, and  % MI-adherent) interrater agreement estimates were also in the excellent range.

### Participant flow

Of the 502 patients approached by researchers, 413 agreed to be screened for eligibility and 92 (22.3%) screened positive for risky drinking. Among ineligible screens, 291 did not report any past year risky drinking, and 5 exceeded the AUDIT-C upper cutoff. Other common reasons for ineligibility were lack of English fluency (n = 15) and pregnancy (n = 3). Of those who screened positive, 45 (48.9%) enrolled in the study: 15 were assigned to NIAAA, 14 were assigned to the BA, and 16 were assigned to MI. The primary reason for non-enrollment was lack of time.

### Participant characteristics

Participants were predominantly female (60%) and 42% were Hispanic. On average, participants reported between 1 and 156 heavy drinking days in the past year (mean 26 (SD = 42)). AUDIT-C scores ranged from 3 to 7 (mean 4.6 (SD = 1.3)).

### Timing

Mean time to complete SBI varied across conditions, with BA taking, on average, 7 min (SD = 6, Range 4–14), NIAAA 9 min (SD = 3, Range 6–18), and MI 17 min (SD = 6, Range 5–32).

### SBI fidelity

Fidelity to intervention components, as measured by the fidelity checklists was high across all conditions. Overall, the clinician completed between 92 and 96% of protocol elements, with little variability within or across conditions. The mean percent fidelity did not differ across BA, NIAAA, and MI, which had respective scores of 96% (SD = 7%), 94% (SD = 6%), and 92% (SD = 6%) (p = 0.32).

### MI differentiability

Significant differences between conditions were found for nearly all MITI constructs (Table 1). All differences were in the expected direction with MI showing the highest doses of MI, then NIAAA, and then BA. The MI condition met or exceeded MI competency standards on all measured domains with the exception of reflection to question ratio. BA and NIAAA did not contain levels of MI that are normally defined as meeting MI competency standards on any of the assessed domains, with the exception of percent complex reflections for the NIAAA condition.

**Table 1:** MITI scores among conditions

Skill	MITI 3.1.1 competency	BA (N = 13)		NIAAA (N = 15)		MI (N = 16)		Pairwise comparisons, p < 0.006*		
		Mean (SD)	99.4% CI+	Mean (SD)	99.4% CI	Mean (SD)	99.4% CI	BA v. NIAAA	BA v. MI	NIAAA v. MI
Evocation	4	1.9 (1.0)	(1.2, 2.5)	3.1 (1.0)	(2.4, 3.7)	4.8 (0.4)	(4.2, 5.4)	Yes	Yes	Yes
Collaboration	4	1.7 (0.9)	(1.1, 2.3)	3.0 (0.9)	(2.5, 3.5)	5.0 (0.0)	(4.5, 5.5)	Yes	Yes	Yes
Autonomy support	4	2.9 (0.5)	(2.5, 3.3)	3.6 (0.7)	(3.2, 4.0)	5.0 (0.0)	(4.6, 5.4)	Yes	Yes	Yes
Direction	4	5.0 (0.0)	–	5.0 (0.0)	–	5.0 (0.0)	–	–	–	–
Empathy	4	1.8 (0.9)	(1.1, 2.4)	2.9 (1.1)	(2.3, 3.5)	5.0 (0.0)	(4.4, 5.6)	Yes	Yes	Yes
Percent open questions	70%	0.0 (0.1)	(− 0.1, 0.1)	0.2 (0.1)	(0.1, 0.3)	0.4 (0.1)	(0.3, 0.4)	Yes	Yes	Yes
Percent complex reflections	50%	0.4 (0.2)	(0.2, 0.5)	0.5 (0.2)	(0.4, 0.6)	0.7 (0.1)	(0.6, 0.8)	No	Yes	No
Reflection to question ratio	2	0.6 (0.2)	(− 0.1, 1.2)	1.0 (1.2)	(0.4, 1.5)	1.5 (0.6)	(0.9, 2.0)	No	No	No
Percent MI adherent	90%	0.1 (0.2)	(− 0.1, 0.3)	0.3 (0.4)	(0.1, 0.4)	1.0 (0.0)	(0.8, 1.2)	No	Yes	Yes

+99.4% confidence intervals with type I error rate Bonferroni-corrected for 9 comparisons (α = 0.006)

*P-values from pairwise, Tukey-adjusted comparisons of least squares mean scores, with additional Bonferroni adjustment for 9 comparisons

## Discussion

In this study, we found that it is possible to conduct high fidelity SBI with differing doses of MI. When implemented with risky drinking primary care patients our three SBI protocols yielded high, moderate, and low doses of MI based on MITI score assessments. This study lays for the groundwork for future research to prospectively compare the efficacy of SBI protocols with varying doses of MI. The protocols can be used in their current form to conduct such work and the MI protocol can provide a standardized MI-based SBI protocol for other work. The availability of these protocols may help to shine light on the unclear picture regarding the role of MI in SBI for risky alcohol use in primary care. Similarly, in the area of treatment and in other settings where the impact of MI structure and content on outcome is somewhat equivocal, the development of highly differentiable interventions may allow for improve prospective and retrospective analysis of effects.

The study has several strengths and limitations. The study sample was all English speaking and result may not be generalizeable to other clinic populations. The methods were tightly controlled resulting in strong internal validity and could be easily translated to a future prospective comparison of the interventions using an efficacy trial design. However, the current study does not necessarily reflect rates of fidelity or MI differentiability that might be observed in an effectiveness trial that involves real-world clinicians. Additionally, training and oversight of the study interventionist was much more intensive than might be feasible with non-research staff.

The protocols in the current study were also primarily conducted by one study interventionist. While this factor eliminates the risk of therapist effects confounding observed differences across SBI protocols, it may limit the generalizability of findings. Set the stage for other research that attempts to dismantle without clearly demonstrating idffereintiability.

Overall, this study represents an important contribution to the research base focused on mechanism of action of SBI for risky alcohol use in primary care. Conducting SBI with high doses of MI takes more time and requires more training and oversight, resulting in many feasibility barriers in the current medical training and practice environment. The current study provides researchers with a tool to address this important question. Future research should seek to compare the efficacy of the three SBI protocols, as well as determine the feasibility of training non-research clinicians to conduct the interventions with fidelity to both structure and MI dose, determine the intensity of training and support needed to yield such results and compare the protocols using a effectiveness trial design.

## Conclusions

 This study demonstrated that it is possible to create protocols for SBI for risky alcohol use that vary in MI dose, while maintaining fidelity to a structured SBI protocol. Fidelity ratings were consistently high and did not vary across the three SBI conditions, while nearly all measures of MI consistency varied across conditions in the expected direction. The only exception was reflection to question ratio, which may have been suppressed because of the screening and assessment questions at the beginning of the protocol. This study suggests that it is possible to unbundle SBI and MI to inform whether varying outcomes relate to the intensity of MI components in SBI protocols. Future studies should investigate potential differential outcomes of SBI protocols that contain no, little, or extensive MI skills and strategies.

## Additional files

**Additional file 1.** NIAAA Clinician's Guide Protocol.

**Additional file 2.** Brief Advice Protocol.

**Additional file 3.** MI-enhanced Clinician's Guide Protocol.

**Additional file 4.** NIAAA Clinician's Guide Fidelity Checklist.

**Additional file 5.** Brief Advice Fidelity Checklist.

**Additional file 6.** MI-enhanced Clinician's Fidelity Checklist.

## Abbreviations

AUD: alcohol use disorders
AUDIT-C: Alcohol Use Disorders Identification Test - Consumption
BA: brief advice (intervention condition)
BI: brief intervention
MI: motivational interviewing
MITI: motivational interviewing treatment integrity code
NIAAA: National Institute on Alcohol Abuse and Alcoholism
SBI: screening and brief intervention
