# Supplementary material for: Development and differentiability of three brief interventions for risky alcohol use that include varying doses of motivational interviewing
Source: Addict Sci Clin Pract. 2018 Feb 27;13:6. doi: 10.1186/s13722-017-0102-0 (PMC5828117; doi:10.1186/s13722-017-0102-0)
Supplement: Supplementary file 2 — Additional file 2. Brief Advice Protocol. [file 13722_2017_102_MOESM2_ESM.pdf]

Brief Advice Clinician's Guide

University of New Mexico
Health Sciences Center
Department of Family & Community Medicine

Prescreen:

“Do you sometimes drink beer, wine, or other alcoholic beverages?”

NO

Screening complete.

YES

Ask the screening question about heavy drinking days:

"How many times in the past year have you had...
...(for men) 5 or more drinks in a day?"
...(for women) 4 or more drinks in a day?"

One standard drink equals 12 oz. of beer, 5 oz of wine, or 1.5 oz. of 80-proof spirits (refer to chart "What's a Standard Drink?").

If the patient has had **any** heavy drinking days in the past year, the screening is positive.

Is the screening positive?

NO

Advise staying within these limits:

"Based on your responses, your drinking falls under maximum drinking limits. I recommend you (continue to) stay within these limits which are:

For healthy men up to age 65 —
• No more than 4 drinks in a day AND
• No more than 14 drinks in a week.

For healthy women (and healthy men over age 65) —
• No more than 3 drinks in a day AND
• No more than 7 drinks in a week.

For pregnant women —
• No alcohol consumption.

For those who have a chronic medical condition exacerbated by alcohol —
• No alcohol consumption.

For those who take medications that interact with alcohol —
• No alcohol consumption.

Advise: "Keep in mind that we are here and open to talking about alcohol use or any (related health) concerns that may arise in the future."

END

YES

Your patient is a risky drinker. For a complete picture of the drinking pattern, determine the weekly average.

"On average, how many days a week do you have an alcoholic drink?"

"On a typical drinking day, how many drinks do you have?"

(Continue on page 4)

If your patient is a risky drinker...

Assess for an alcohol use disorder by asking the following DSM-IV 2-Item Scale Questions:

"In the past 12 months, have there often been times when you had a lot more to drink than you intended to have?"	☐ **YES**	☐ **NO**
"In the past 12 months, have you sometimes been under the influence of alcohol in situations where you could have caused an accident or gotten hurt?"	☐ **YES**	☐ **NO**

- If negative to both DSM-IV 2-Item Scale questions, the patient is unlikely to have an AUD. Move on to assessing for medication or medical complications of use.
- If positive to one of the DSM-IV 2-Item Scale questions, assess patient for other AUD criteria.
- If positive to both DSM-IV 2-Item Scale questions, the patient meets criteria for AUD by DSM-5. Assess further for additional AUD criteria to determine severity.
- If patient has voluntarily or spontaneously reported symptoms up to this point or during assessment, record them.
- If specific assessment questions seem particularly appropriate given the patient's presentation, use those first.

Assess elements of an Alcohol Use Disorder (AUD)

Ask the following questions: *"In the past 12 months...*

Continued use despite recurrent interpersonal or social problems

...have you continued to drink even though you knew it was causing you trouble with your family or friends, or have you gotten into physical altercations while drinking or right after drinking?" ☐ YES ☐ NO

Failure to fulfill major role obligations at work, school, or home due to recurrent drinking

...have you had a period when your drinking (or being sick from drinking) often interfered with taking care of your home or family, or caused problems with work or school?" ☐ YES ☐ NO

Craving, strong desire or urge to use alcohol

...have you had such a strong desire to drink that it was difficult to think of anything else?" ☐ YES ☐ NO

Impaired control

...have you more than once wanted to stop or cut down on your drinking, or tried more than once to stop or cut down but found you couldn't?" ☐ YES ☐ NO

Tolerance

...have you found that you have to drink much more than you once did to get the effect you want, or that your usual number of drinks has much less effect on you than it once did?" ☐ YES ☐ NO

Withdrawal syndrome or drinking to relieve withdrawal

...when the effects of alcohol are wearing off, have you had trouble sleeping, found yourself shaking, nervous, nauseous, restless, sweating or with your heart beating fast, sensed things that aren't really there, or had seizures?" ☐ YES ☐ NO

...have you taken a drink or used any drug or medicine (other than over-the-counter pain relievers) to keep from having bad aftereffects of drinking, or to get over them?" ☐ YES ☐ NO

Continued use despite recurrent psychological or physical problems

...have you continued to drink even though you knew it was making you feel depressed or anxious, causing a health problem or making one worse, or following a blackout?" ☐ YES ☐ NO

Time spent related to drinking or recovering

...have you had a period when you spent a lot of time drinking, being sick, or recovering from the aftereffects of drinking? ☐ YES ☐ NO

Neglect of activities

...in order to drink, have you given up or cut down on activities that were important or interesting to you or gave you pleasure?" ☐ YES ☐ NO

- **AUD Thresholds**: If 2-3 positive = mild AUD (use clinical judgment, you can go down either risky drinking or AUD path). If positive to 4 or more criteria above, use AUD Route.

Does the patient meet criteria for an alcohol use disorder (AUD)?

YES

Your patient has an alcohol use disorder.

State your conclusions and recommendations clearly:

"I believe that you have an alcohol use disorder. I recommend that you quit drinking. Abstaining is the safest course for most patients with alcohol use disorders."

"You may benefit from seeing a provider who specializes in addictions and we have some available in this clinic that I could refer you to. There are also services that are available in the community. We could also talk with your doctor about monitoring you or exploring medications that might be helpful."

"Which of these options would you like to pursue?"

Make appropriate referrals or provide relevant information using Strategies for Cutting Down.

If not ready, acknowledge that change is difficult and state: "I'd encourage you to contact your provider if you decide you'd like any help with alcohol."

If patient asks for clarification or factual information, answer and/or repeat information provided above. If patient indicates an interest in exploring his/her views on the topic, suggest referral to doctor or behavioral health.

NO

Your patient is still at risk for developing alcohol-related problems.

State your conclusions and recommendations clearly:

Refer to chart "U.S. Adult Drinking Patterns".

"Your replies indicate that your alcohol consumption exceeds the maximum drinking limits. As you can see from this sheet, women (men) are advised to drink no more than 7 (14) per week, and 3 (4) per occasion. As you see in this chart, 72% of people never exceed these limits. High alcohol consumption can increase your chances of developing an alcohol use disorder. If your alcohol consumption exceeds the maximum drinking limit you may also experience a number of adverse consequences including accidents or problems with your family, work, education, or finances. You may also experience harmful health effects such as damage to your liver, gastrointestinal diseases, high blood pressure, and cancers."

"Based on this, I recommend that you cut down and I'm willing to help."

"If you decide you want to make a change, here are some tips."

Paraphrase Strategies for Cutting Down.

"You can also contact your doctor or a behavioral health specialist at the clinic using the contact information provided here."

"Do you have any questions for me?"

If patient asks for clarification or factual information, answer and/or repeat information provided above. If patient has brief response, simple reflections can be provided. If patient indicates an interest in exploring his/her views on the topic in depth, suggest referral to doctor or behavioral health.
