# Supplementary material for: Development and differentiability of three brief interventions for risky alcohol use that include varying doses of motivational interviewing
Source: Addict Sci Clin Pract. 2018 Feb 27;13:6. doi: 10.1186/s13722-017-0102-0 (PMC5828117; doi:10.1186/s13722-017-0102-0)
Supplement: Supplementary file 3 — Additional file 3. MI-enhanced Clinician's Guide Protocol. [file 13722_2017_102_MOESM3_ESM.pdf]

MI-Enhanced Clinician's Guide

University of New Mexico
Health Sciences Center
Department of Family &
Community Medicine

"In medical settings, we commonly talk with patients about alcohol consumption. Would it be all right if we spend a few minutes talking about alcohol?"

Prescreen:

"Do you sometimes drink beer, wine, or other alcoholic beverages?"

NO

Screening complete.

YES

Ask the screening question about heavy drinking days:

"How many times in the past year have you had...
...(for men) 5 or more drinks in a day?"
...(for women) 4 or more drinks in a day?"

One standard drink equals 12 oz. of beer, 5 oz of wine, or 1.5 oz. of 80-proof spirits (refer to chart "What's a Standard Drink?").

If the patient has had **any** heavy drinking days in the past year, the screening is positive.

Is the screening positive?

NO

Advise staying within these limits:

"Based on your responses, your drinking falls under maximum drinking limits. I recommend you (continue to) stay within these limits which are:

For healthy men up to age 65 —
- No more than 4 drinks in a day AND
- No more than 14 drinks in a week.

For healthy women (and healthy men over age 65) —
- No more than 3 drinks in a day AND
- No more than 7 drinks in a week.

For pregnant women —
- No alcohol consumption.

For those who have a chronic medical condition exacerbated by alcohol —
- No alcohol consumption.

For those who take medications that interact with alcohol —
- No alcohol consumption.

Advise: "Keep in mind that we are here and open to talking about alcohol use or any (related health) concerns that may arise in the future."

END

YES

Your patient is a risky drinker. For a complete picture of the drinking pattern, determine the weekly average.

"Great. Could you tell me a little more about your drinking patterns?"

Reflect.

If needed, follow up with specific questions:

"On average, how many days a week do you have an alcoholic drink?"

"On a typical drinking day, how many drinks do you have?"

Reflect.

Assess for an alcohol use disorder:

"Thinking about the past year, what have been some of the drawbacks of drinking, if anything?"

Reflect.

If patient has voluntarily or spontaneously reported symptoms up to this point or during assessment, record them.

Next, say: "If it's all right, I have a few more specific questions that I'd like to ask, because they can help us pinpoint where you are in terms of drinking."

Ask the following DSM-IV 2-Item Scale Questions:

"In the past 12 months, have there often been times when you had a lot more to drink than you intended to have?"	☐ **YES**	☐ **NO**
"In the past 12 months, have you sometimes been under the influence of alcohol in situations where you could have caused an accident or gotten hurt?"	☐ **YES**	☐ **NO**

- If <u>negative</u> to both DSM-IV 2-Item Scale questions, the patient is unlikely to have an AUD. Move on to assessing for medication or medical complications of use.
- If <u>positive to one</u> of the DSM-IV 2-Item Scale questions, assess patient.
- If <u>positive to both</u> DSM-IV 2-Item Scale questions, the patient meets criteria for AUD by DSM-5. Assess further for additional AUD criteria to determine severity.

Assess elements of an Alcohol Use Disorder (AUD)

Ask the following questions:

Reflect on patient's responses, when appropriate, and attend to change talk.

"In the past 12 months...

Continued use despite recurrent interpersonal or social problems

...have you continued to drink even though you knew it was causing you trouble with your family or friends, or have you gotten into physical altercations while drinking or right after drinking?" ☐ YES ☐ NO

Failure to fulfill major role obligations at work, school, or home due to recurrent drinking

...have you had a period when your drinking (or being sick from drinking) often interfered with taking care of your home or family, or caused problems with work or school?" ☐ YES ☐ NO

Craving, strong desire or urge to use alcohol

...have you had such a strong desire to drink that it was difficult to think of anything else?" ☐ YES ☐ NO

Impaired control

...have you more than once wanted to stop or cut down on your drinking, or tried more than once to stop or cut down but found you couldn't?" ☐ YES ☐ NO

Tolerance

...have you found that you have to drink much more than you once did to get the effect you want, or that your usual number of drinks has much less effect on you than it once did?" ☐ YES ☐ NO

Withdrawal syndrome or drinking to relieve withdrawal

...when the effects of alcohol are wearing off, have you had trouble sleeping, found yourself shaking, nervous, nauseous, restless, sweating or with your heart beating fast, sensed things that aren't really there, or had seizures?" ☐ YES ☐ NO

...have you taken a drink or used any drug or medicine (other than over-the-counter pain relievers) to keep from having bad aftereffects of drinking, or to get over them?" ☐ YES ☐ NO

Continued use despite recurrent psychological or physical problems

...have you continued to drink even though you knew it was making you feel depressed or anxious, causing a health problem or making one worse, or following a blackout?" ☐ YES ☐ NO

Time spent related to drinking or recovering

...have you had a period when you spent a lot of time drinking, being sick, or recovering from the aftereffects of drinking? ☐ YES ☐ NO

Neglect of activities

...in order to drink, have you given up or cut down on activities that were important or interesting to you or gave you pleasure?" ☐ YES ☐ NO

- **AUD Thresholds**: If 2-3 positive = mild AUD (use clinical judgment, you can go down either risky drinking or AUD path). If positive to 4 or more criteria above, use AUD Route.

Does the patient meet criteria for an alcohol use disorder (AUD)?

YES

Your patient has an alcohol use disorder.

“Thanks for taking the time to talk with me about your alcohol use. Would it be all right if I share some feedback about your drinking?”

“What are your thoughts about how a doctor might classify you in terms of your drinking?”

Reflect on response.

“Would it be all right if I shared some information with you about this?”

“Based on your responses it appears you may have what doctors refer to as an Alcohol Use Disorder. This means alcohol use is interfering with important areas of your life. It can be harder for people with alcohol use disorders to moderate their drinking. We also know that people with alcohol use disorders often find it helpful to talk with a counselor about their drinking.”

“What do you make of all that?”

Reflect.

“Where does this leave you?”

NO

Your patient is still at risk for developing alcohol-related problems.

“Thanks for taking the time to talk with me about your alcohol use. Would it be all right if I share some information about safe drinking limits?”

“What do you know about what doctors recommend as maximum drinking limits and what some of the health risks of exceeding these limits are?”

Reflect on response.

“Would it be all right if I shared some additional information with you from this chart?”

Refer to chart “U.S. Adult Drinking Patterns”.

Share information in tailored way.

“What concerns you most with this chart?”

Reflect.

“Where does this leave you?”

Is your patient ready to make changes / reduce drinking?

NO

Build motivation using readiness ruler, road map, or decisional balance.

- Ask permission
- Explain
- Evoke
- Reflect
- Ask where does this leave you?

Reflect.

Transition to action-planning if appropriate.

Otherwise, say: "Thanks for taking the time to talk with me and explore these issues. I appreciate your honesty and self-reflection. If you decide in the future that this is an issue that you'd like to revisit, what ideas do you have about how you might be able to reach out to a provider?"

Is your (AUD) patient ready to accept referral?

YES

"What do you know about resources that are available to help people who are thinking about making changes in their drinking?"

Share referral information if appropriate.

Is your risky drinking patient ready to make changes / reduce drinking?

YES

"What kinds of changes are you hoping to make to your drinking?"

"What ideas do you have about how you might go about that?"

Share Strategies for Cutting Down if appropriate.

"Which ones stand out to you as strategies that might be helpful?"

Summarize reasons for change and plan.

State:
"Thanks for taking the time to talk with me and explore these issues.
I appreciate your honestly and self-reflection."
