# Supplementary material for: Development and differentiability of three brief interventions for risky alcohol use that include varying doses of motivational interviewing
Source: Addict Sci Clin Pract. 2018 Feb 27;13:6. doi: 10.1186/s13722-017-0102-0 (PMC5828117; doi:10.1186/s13722-017-0102-0)
Supplement: Supplementary file 4 — Additional file 4. NIAAA Clinician's Guide Fidelity Checklist. [file 13722_2017_102_MOESM4_ESM.pdf]

NIAAA Fidelity Checklist

Participant ID: ______________________ **Therapist:** ______________________________

Rater: ____ **Date:** ____________________

SCREENING

**NIAAA Tasks** *(Indicate whether therapist completes each task)*		**Notes**
Prescreen: Do you sometimes drink beer, wine, or other alcoholic beverages? ***(If NO, screening is complete.)***	☐ YES ☐ NO	
Single Question Screen: How many times in the past year have you had 5 (4 for women) or more drinks in a day?	☐ YES ☐ NO	
Gives standard drink description (12oz-beer, 5oz-wine, 1.5oz-80 proof liquor)	☐ YES ☐ NO	

Is the screening positive? NO

NIAAA Fidelity Checklist

<u>WITHIN</u> RECOMMENDED LIMITS ☐ (NOT APPLICABLE)

For patients drinking within recommended limits, advise they continue to stay within these limits:	Notes

"Based on your responses, your drinking falls under maximum drinking limits. I recommend you (continue to) stay within these limits which are..." .... ☐ YES ☐ NO

For healthy men up to age 65: no more than 4 drinks in a day AND no more than 14 drinks in a week.

OR

For healthy women (and healthy men over age 65): no more than 3 drinks in a day AND no more than 7 drinks in a week. .... ☐ YES ☐ NO

For pregnant women: no alcohol consumption. .... ☐ YES ☐ NO

☐ NOT APPLICABLE

For those who have a chronic medical condition exacerbated by alcohol: no alcohol consumption. .... ☐ YES ☐ NO

For those who take medications that interact with alcohol: no alcohol consumption.................................. ☐ YES ☐ NO

Advise: "Keep in mind that we are here and open to talking about alcohol use or any (related health) concerns that may arise in the future."................................. ☐ YES ☐ NO

Is the screening positive? YES

NIAAA Fidelity Checklist

MORE THAN RECOMMENDED LIMITS ☐ (NOT APPLICABLE)

For patients drinking more than recommended limits. *(Scaling Questions)*	Notes
Assess Risk:<br>**Frequency** (how many days in a week?) ☐ YES ☐ NO	
Assess Risk:<br>**Quantity** (how many drinks per day?) ☐ YES ☐ NO	
Assess for Alcohol Use Disorder (AUD):<br>Abuse, Dependence **(Drinking more than intended)** ☐ YES ☐ NO	
Assess for AUD:<br>Abuse, Dependence **(Risk bodily harm)** ☐ YES ☐ NO	
Assess AUD further if either of the last 2 questions was positive, NA if last two questions were negative. ☐ YES ☐ NO<br>☐ NOT APPLICABLE	

NIAAA Fidelity Checklist

BRIEF INTERVENTION

AUD Positive ☐ (NOT APPLICABLE)

State "I believe you have an AUD." .... ☐ YES ☐ NO

"I recommend you quit drinking and I'm willing to help." .... ☐ YES ☐ NO

Relate to patient's concerns and medical findings if present. .... ☐ YES ☐ NO
☐ NOT APPLICABLE

State "Let's come up with a plan. Abstaining is the safest course for most patients with AUDs." .... ☐ YES ☐ NO

Explain referral options.......................... ☐ YES ☐ NO

Ask "Which of these options would you like to pursue?"...................... ☐ YES ☐ NO

Make appropriate referrals or provide Strategies for Cutting Down sheet. .... ☐ YES ☐ NO

Acknowledge that change is difficult and encourage follow-up. .... ☐ YES ☐ NO

AUD Negative ☐ (NOT APPLICABLE)

State you are drinking more than is medically safe. .... ☐ YES ☐ NO

State recommended drinking limits .... ☐ YES ☐ NO

State 72% of (or most) adults never exceed these limits....................... ☐ YES ☐ NO

State risk for developing AUD .... ☐ YES ☐ NO

Daily consequences (accidents, family, education, work, money problems) .... ☐ YES ☐ NO

Weekly consequences (liver, gastrointestinal disease, high blood pressure, cancers) .... ☐ YES ☐ NO

State "I recommend you cut down and I'm willing to help."................. ☐ YES ☐ NO

Ask "Are you willing to consider making changes to your drinking?" .... ☐ YES ☐ NO

For risky drinking patients, are they ready to make a change?

NIAAA Fidelity Checklist

READINESS

Not Ready ☐ (NOT APPLICABLE)

State "Ok, I'd like to restate that I am concerned about your health." .... ☐ YES ☐ NO

State "Tell me a little about what you like about drinking versus your reasons for cutting down." .... ☐ YES ☐ NO

State, "Ok, what are the major barriers to change?" .... ☐ YES ☐ NO

State "Great, if you decide to change, you can contact the behavioral health specialist at the clinic." .... ☐ YES ☐ NO

State "Keep in mind that your doctor is here and willing to help if you decide you want to make a change." .... ☐ YES ☐ NO

Ready ☐ (NOT APPLICABLE)

"Great. Let's set a goal to cut down within maximum limits." .... ☐ YES ☐ NO

State "We'll agree on a plan regarding what specific steps you are going to take." .... ☐ YES ☐ NO

"So let's think about:

- What specific steps are you going to take? .... ☐ YES ☐ NO
- How are you going to track your drinking? .... ☐ YES ☐ NO
- How are you going to manage high risk situations? .... ☐ YES ☐ NO
- Who might be willing to help you?" ...... ☐ YES ☐ NO

Allow response. .... ☐ YES ☐ NO

Give Strategies for Cutting Down sheet and paraphrase. .... ☐ YES ☐ NO

State "You can check some off to try the first week and add some others the next week.Which would you like to try first?" .... ☐ YES ☐ NO

State "Great, I encourage you to follow-up with your doctor with questions ... . ☐ YES ☐ NO

…or if you have trouble sticking to your plan" .... ☐ YES ☐ NO

NIAAA Fidelity Checklist

FIDELITY INCONSISTENCIES
(LIST STATEMENTS OR QUESTIONS THAT VEER FROM THE PROTOCOL)

1

2

3

4

5

Total Inconsistencies: ____________________
