# Supplementary material for: Development and differentiability of three brief interventions for risky alcohol use that include varying doses of motivational interviewing
Source: Addict Sci Clin Pract. 2018 Feb 27;13:6. doi: 10.1186/s13722-017-0102-0 (PMC5828117; doi:10.1186/s13722-017-0102-0)
Supplement: Supplementary file 5 — Additional file 5. Brief Advice Fidelity Checklist. [file 13722_2017_102_MOESM5_ESM.pdf]

Brief Advice Fidelity Checklist

Participant ID: ______________________ **Therapist:** ______________________

Rater: ______________________ **Date:** ______________________

SCREENING

Brief Advice Tasks *(Indicate whether therapist completes each task)*	Notes
<u>Prescreen</u>: Do you sometimes drink beer, wine, or other alcoholic beverages? ***(If NO, screening is complete)*** .... ☐ YES ☐ NO	
<u>Single Question Screen</u>: How many times in the past year have you had 5 (4 for women) or more drinks in a day? .... ☐ YES ☐ NO	
Gives standard drink description (12oz-beer, 5oz-wine, 1.5oz-80 proof liquor) .... ☐ YES ☐ NO	

Is the ISA screening positive? NO

Brief Advice Fidelity Checklist

WITHIN RECOMMENDED LIMITS ☐ (NOT APPLICABLE)

For patients drinking within recommended limits, advise they continue to stay within these limits:	Notes
"Based on your responses, your drinking falls under maximum drinking limits. I recommend you (continue to) stay within these limits which are..." .... ☐ YES ☐ NO	
For healthy men up to age 65: no more than 4 drinks in a day AND no more than 14 drinks in a week.<br>**OR**<br>For healthy women (and healthy men over age 65): no more than 3 drinks in a day AND no more than 7 drinks in a week. .... ☐ YES ☐ NO	
For pregnant women: no alcohol consumption. .... ☐ YES ☐ NO<br>☐ NOT APPLICABLE	
For those who have a chronic medical condition exacerbated by alcohol: no alcohol consumption. .... ☐ YES ☐ NO	
For those who take medications that interact with alcohol: no alcohol consumption.................................. ☐ YES ☐ NO	
**Advise**: "Keep in mind that we are here and open to talking about alcohol use or any (related health) concerns that may arise in the future.".................................. ☐ YES ☐ NO	

Is the screening positive? YES

Brief Advice Fidelity Checklist

MORE THAN RECOMMENDED LIMITS ☐ (NOT APPLICABLE)

**For patients drinking more than recommended limits.** *(Screening Questions)*	**Notes**
Assess Risk:<br>**Frequency** (how many days in a week?) .... ☐ YES ☐ NO	
Assess Risk:<br>**Quantity** (how many drinks per day?) .... ☐ YES ☐ NO	
Assess for Alcohol Use Disorder (AUD):<br>Abuse, Dependence **(Drinking more than intended)** .... ☐ YES ☐ NO	
Assess for AUD:<br>Abuse, Dependence **(Risk bodily harm)** .... ☐ YES ☐ NO	
Assess AUD further if either of the last 2 questions was positive, NA if last two questions were negative. .... ☐ YES ☐ NO<br>☐ NOT APPLICABLE	

Brief Advice Fidelity Checklist

BRIEF INTERVENTION

AUD Positive ☐ (NOT APPLICABLE)

State "I believe you have an AUD."	☐ YES	☐ NO
"I recommend you quit drinking."	☐ YES	☐ NO
"Abstaining is the safest course for most patients with AUDs."	☐ YES	☐ NO
Explain referral options.	☐ YES	☐ NO
Ask "Which of these options would you like to pursue?"	☐ YES	☐ NO
Make appropriate referrals or state strategies for cutting down.	☐ YES	☐ NO
Encourage follow-up.	☐ YES	☐ NO

AUD Negative ☐ (NOT APPLICABLE)

State "Based on your responses, you are, your alcohol consumption exceeds the maximum recommended drinking limits"	☐ YES	☐ NO
State recommended drinking limits	☐ YES	☐ NO
State 72% of (or most) adults never exceed these limits	☐ YES	☐ NO
Daily consequences (accidents, family, education, work, money problems)	☐ YES	☐ NO
Weekly consequences (liver, gastrointestinal disease, high blood pressure, cancers)	☐ YES	☐ NO
State risk for developing AUD	☐ YES	☐ NO
State "I recommend you cut down and I'm willing to help."	☐ YES	☐ NO
Give Strategies for Cutting Down sheet and summarize the sheet.	☐ YES	☐ NO
Give referral information.	☐ YES	☐ NO
Ask if they have questions	☐ YES	☐ NO

Brief Advice Fidelity Checklist

FIDELITY INCONSISTENCIES
(LIST STATEMENTS OR QUESTIONS THAT VEER FROM THE PROTOCOL)

1

2

3

4

5

Total Inconsistencies: ____________________
