# Supplementary material for: Development and differentiability of three brief interventions for risky alcohol use that include varying doses of motivational interviewing
Source: Addict Sci Clin Pract. 2018 Feb 27;13:6. doi: 10.1186/s13722-017-0102-0 (PMC5828117; doi:10.1186/s13722-017-0102-0)
Supplement: Supplementary file 6 — Additional file 6. MI-enhanced Clinician's Fidelity Checklist. [file 13722_2017_102_MOESM6_ESM.pdf]

MI-Enhanced Fidelity Checklist

Participant ID: ______________________ **Therapist:** ______________________

Rater: ______________________ **Date:** ______________________

SCREENING

**Motivational Interviewing Tasks** *(Indicate whether therapist completes each task)*			**Notes**
State "In medical settings, we commonly talk with patients about alcohol consumption.	☐ YES	☐ NO	
Would it be all right if we spend a few minutes talking about alcohol?"	☐ YES	☐ NO	
Prescreen: Do you sometimes drink beer, wine, or other alcoholic beverages? ***(If NO, screening is complete.)***	☐ YES	☐ NO	
Single Question Screen: How many times in the past year have you had 5 (4 for women) or more drinks in a day?	☐ YES	☐ NO	
Gives standard drink description (12oz-beer, 5oz-wine, 1.5oz-80 proof liquor)	☐ YES	☐ NO	

If the patient has had ANY heavy drinking days in the past year, the screen is positive. **Continue**

MI-Enhanced Fidelity Checklist

WITHIN RECOMMENDED LIMITS ☐ (NOT APPLICABLE)

For patients drinking within recommended limits, advise they continue to stay within these limits:			Notes
"Based on your responses, your drinking falls under maximum drinking limits. I recommend you (continue to) stay within these limits which are..."	☐ YES	☐ NO	
For healthy men up to age 65: no more than 4 drinks in a day AND no more than 14 drinks in a week.<br>**OR**<br>For healthy women (and healthy men over age 65): no more than 3 drinks in a day AND no more than 7 drinks in a week.	☐ YES	☐ NO	
For pregnant women: no alcohol consumption.<br>☐ NOT APPLICABLE	☐ YES	☐ NO	
For those who have a chronic medical condition exacerbated by alcohol: no alcohol consumption.	☐ YES	☐ NO	
For those who take medications that interact with alcohol: no alcohol consumption.	☐ YES	☐ NO	
**Advise**: "Keep in mind that we are here and open to talking about alcohol use or any (related health) concerns that may arise in the future."	☐ YES	☐ NO	

Is the screening positive? YES

MI-Enhanced Fidelity Checklist

MORE THAN RECOMMENDED LIMITS ☐ (NOT APPLICABLE)

For patients drinking more than recommended limits. *(Screening Questions)*			Notes
State "Great, could you tell me a little more about your drinking pattern?"	☐ YES	☐ NO	
Reflect answer to "could you tell me more about your drinking?"	☐ YES	☐ NO	
If needed, ask "On average, how many days a week do you have an alcoholic drink?"<br>☐ NOT APPLICABLE	☐ YES	☐ NO	
If needed, ask "On a typical day, how many drinks do you have?"<br>☐ NOT APPLICABLE	☐ YES	☐ NO	
Reflect.	☐ YES	☐ NO	
Ask "Thinking about the past year, what have been some of the drawbacks of drinking, if anything?"	☐ YES	☐ NO	
Reflect.	☐ YES	☐ NO	
If needed, assess for Alcohol Use Disorder (AUD): Ask permission and headline about more questions that help us pinpoint.<br>☐ NOT APPLICABLE	☐ YES	☐ NO	
If needed, assess for Alcohol Use Disorder (AUD): Abuse, Dependence **(Drinking more than intended)**<br>☐ NOT APPLICABLE	☐ YES	☐ NO	
If needed, assess for AUD: Abuse, Dependence **(Risk bodily harm)**<br>☐ NOT APPLICABLE	☐ YES	☐ NO	
Assess AUD further if either of the last 2 questions was positive, NA if last two questions were negative.<br>☐ NOT APPLICABLE	☐ YES	☐ NO	

MI-Enhanced Fidelity Checklist

BRIEF INTERVENTION

AUD Positive ☐ (NOT APPLICABLE)

State "Thanks for taking the time to talk with me about your alcohol use. Would it be all right if I share some feedback about your drinking?................ ☐ YES ☐ NO

Ask "What are your thoughts about how a doctor might classify you in terms of your drinking?" .... ☐ YES ☐ NO

Reflect.. ☐ YES ☐ NO

"Would it be all right if I shared some information with you about this?"........... ☐ YES ☐ NO

"Based on your responses it appears you may have what doctors refer to as an Alcohol Use Disorder. This means alcohol use is interfering with important areas of your life. It can be harder for people with alcohol use disorders to moderate their drinking. We also know that people with alcohol use disorders often find it helpful to talk with a counselor about their drinking." .... ☐ YES ☐ NO

"What do you make of all that?" .... ☐ YES ☐ NO

Reflect.. ☐ YES ☐ NO

Ask "Where does this leave you?" .... ☐ YES ☐ NO

Reflect.. ☐ YES ☐ NO

AUD Negative ☐ (NOT APPLICABLE)

State: "Thanks for taking the time to talk with me about your alcohol use. Would it be all right if I share some information about safe drinking limits?" .... ☐ YES ☐ NO

Ask "What do you know about what doctors recommend as maximum drinking limits and what some of the health risk of exceeding these limits are?" .... ☐ YES ☐ NO

Reflect. .... ☐ YES ☐ NO

"Would it be all right if I shared some additional information with you from this chart?" .... ☐ YES ☐ NO

Refer to chart "U.S. Adult Drinking Patterns"; share information in a tailored way. .... ☐ YES ☐ NO

"What concerns you most with this chart?" .... ☐ YES ☐ NO

Reflect. .... ☐ YES ☐ NO

Ask "Where does this leave you?"........... ☐ YES ☐ NO

Reflect.. ☐ YES ☐ NO

MI-Enhanced Fidelity Checklist

READINESS

Not Ready ☐ (NOT APPLICABLE)

Build motivation using readiness ruler, road map, or decisional balance

- Ask Permission ☐ YES ☐ NO
- Explain ☐ YES ☐ NO
- Evoke ☐ YES ☐ NO
- Reflect ☐ YES ☐ NO
- Ask Where Leave ☐ YES ☐ NO

Reflect. ☐ YES ☐ NO

If appropriate:
Transition to action planning ☐ YES ☐ NO
☐ NOT APPLICABLE

Otherwise, say: "Thanks for taking the time to talk with me and explore these issues. I appreciate your honesty and self-reflection... ☐ YES ☐ NO
☐ NOT APPLICABLE

...If you decide in the future that this is an issue that you'd like to revisit, what ideas do you have about how you might be able to reach out to a provider?" ☐ YES ☐ NO
☐ NOT APPLICABLE

Ready ☐ (NOT APPLICABLE)

Ask "What kinds of changes are you hoping to make to your drinking?" ☐ YES ☐ NO

Ask "What ideas do you have about how you might go about that?" ☐ YES ☐ NO

Share Strategies for Cutting Down if appropriate ☐ YES ☐ NO
☐ NOT APPLICABLE

Ask "Which strategies stand out as ones that might be helpful?" ☐ YES ☐ NO
☐ NOT APPLICABLE

(FOR AUD patients)
"What do you know about resources that are available to help people who are thinking about making changes in their drinking?" ☐ YES ☐ NO
☐ NOT APPLICABLE

(FOR AUD patients) Share referral information if appropriate. ☐ YES ☐ NO
☐ NOT APPLICABLE

Summarize the reasons for change and the plan. ☐ YES ☐ NO

State "Thanks for taking the time to talk with me and explore these issues. I appreciate your honesty and self-reflection. ☐ YES ☐ NO

MI-Enhanced Fidelity Checklist

FIDELITY INCONSISTENCIES
(LIST STATEMENTS OR QUESTIONS THAT VEER FROM THE PROTOCOL)

1

2

3

4

5

Total Inconsistencies: ____________________
